# Suicidal Behaviour among School-Going Adolescents in Saint Lucia: Analysis of Prevalence and Associated Factors

**DOI:** 10.3390/bs13070535

**Published:** 2023-06-27

**Authors:** Jacob Owusu Sarfo, Mustapha Amoadu, Paul Obeng, Newton Isaac Gbordzoe, Timothy Pritchard Debrah, Crescens Osei Bonsu Ofori, John Elvis Hagan

**Affiliations:** 1 Department of Health, Physical Education and Recreation, University of Cape Coast, Cape Coast PMB TF0494, Ghana; 2 School of Public Health, University of Ghana, Legon P.O. Box LG 13, Ghana; 3 Department of Nursing, Kwame Nkrumah University of Science and Technology, Private Mail Bag, University Post Office, Kumasi 00233, Ghana; 4 Department of Psychology, University of Ghana, Legon P.O. Box LG 13, Ghana; 5 Neurocognition and Action-Biomechanics-Research Group, Faculty of Psychology and Sports Science, Bielefeld University, Postfach 10 01 31, 33501 Bielefeld, Germany

**Keywords:** suicidal behaviour, suicidal ideation, suicidal planning, suicide attempt, self-injury, adolescents, prevalence, interpersonal theory of suicide, demoralisation, Saint Lucia

## Abstract

Suicide poses a debilitating threat to adolescents’ lives worldwide. Although suicide prevention efforts are evident globally, there is limited evidence on the prevalence and correlations of suicidal behaviour among school-going adolescents in Saint Lucia. We used a dataset from the 2018 Global School-based Student Health Survey to examine the prevalence and associated factors of suicidal behaviour among 1864 students from schools in Saint Lucia. Prevalence rates of 25.5%, 22.1%, and 17.5% were found for suicidal ideation, suicide plan, and suicide attempt, respectively. After adjusting for other factors, being male and having understanding parents were protective against suicidal behaviour. However, suicidal ideation was predicted by being physically attacked and bullied, parental guidance, tobacco use, loneliness, and worry. Moreover, being a victim of physical attacks and bullying, having close friends, being lonely, and worrying were predictive of making suicidal plans among adolescents. Attempting suicide was predicted by cigarette smoking, current use of tobacco and related products, bullying, having close friends, being lonely, and worrying. School-based preventive interventions are required to help address triggers of suicidal behaviour among adolescents in Saint Lucia and to help attain the targets for suicide prevention in the global Sustainable Development Goals.

## 1. Introduction

Globally, about 703,000 people take their own life every year [1]. Moreover, for every suicide, there are many more people who attempt suicide [2]. Suicidal deaths are considered tragedies as they affect families, communities, and societies and have long-lasting mental health effects on relatives and loved ones left behind. It is worth noting that suicide occurs throughout the lifespan. In addition, suicide is a global phenomenon and does not only occur in high-income countries. Generally, the three non-fatal suicidal behaviours that have been researched the most frequently are suicide ideation (SI), suicide planning (SP), and suicide attempt (SA) [2]. Though SI is more common, it is commonly believed to precede SP and SA [3]. In addition, SA is the most critical associated factor for suicidal deaths globally and among young people [1].

Evidence shows that about 77% of global suicide cases in 2019 were reported in low- and middle-income countries (LMICs), with adolescents among the worst-affected groups [1]. Adolescents are highly vulnerable to mental health issues [4,5]. Though most adolescents have good mental health, social, physical, and emotional changes that occur during adolescence may make them vulnerable to mental health issues. Furthermore, societal pressures such as poverty, physical and sexual violence, harsh parenting, and bullying can make adolescents extremely vulnerable to mental health disorders. Moreover, suicide is the fourth leading cause of death among older adolescents (15–19-year-olds) [1].

Furthermore, previous studies have explored the prevalence of suicidal behaviour among adolescents in LMIC using the Global School-based Student Health Survey (GHSH). For instance, a study that examined suicidal behaviour among adolescents from 59 LMICs using GHSH data from 2003 to 2015 reported the highest prevalence of SI and SP in the African region at 20.4% and 23.7%, respectively. In contrast, the Western Pacific had the highest prevalence of SA at 20.5% [6]. However, Uddin et al. did not report on correlates of suicidal behaviour among adolescents in their study. 

### 1.1. Theoretical Framework: Interpersonal Theory of Suicide

Some theories have highlighted the significance of intrapersonal and interpersonal factors in predicting suicidal behaviour. For example, the Interpersonal Theory of Suicide (ITS) [7] may serve as a useful framework for studying the potential pathways to adolescent suicidal behaviour. The ITS [7] hypothesises that two dimensions (i.e., thwarted belongingness and perceived burdensomeness) ought to be present in a potential suicidal behaviour. Thwarted belongingness denotes an absence of or an interruption in social relationships (i.e., low-income family climate, social alienation), which has been hypothesised to be a key stressor or a predictor of suicide [7,8]. The latter dimension, perceived burdensomeness (i.e., distress from liability), mirrors the perspective that one’s existence is a burden on significant others such as family members, friends, and society as a whole. Hence, dying appears more reasonable in the mind of the anguished person [7]. These two schools of thought are purported to trigger suicidal behaviour through issues such as previous self-harm or injury, abusive childhood experiences (e.g., being bullied), and having other risk-taking encounters (e.g., substance use, truancy) in life. Previous empirical inquiries have established associations between different elements of thwarted belongingness and perceived burdensomeness, and suicidal behaviour across different cohorts [9,10,11]. For instance, Hong [12] examined the association between interpersonal needs, family dysfunction, and suicidal behaviour in Korean adolescents and noted that the link between adolescent emotional and physical abuse and SI was significantly mediated by low belongingness. 

### 1.2. The Present Study

Saint Lucia, a small island in the Caribbean, has approximately 184,401 (in 2021) people living on 238 square miles. This island has an urban population of 18.6% (34,141 people in 2020) with a median age of 34.5 years. About 32% of young people in Saint Lucia, aged 10 to 24, are in education. The suicide mortality rate per 100,000 population in Saint Lucia, according to statistics from the World Bank, was 7.40 in 2016, 7.90 in 2017, 8.00 in 2018, and 7.90 in 2019. Though there is limited evidence on the suicidal behaviour of adolescents in Saint Lucia, a recent study on alcohol misuse prevalence and correlates in two Caribbean countries (Saint Lucia and Saint Vincent and the Grenadines) reported SI, SP, and SA prevalence rates of 25%, 21.4%, and 16.8%, respectively, among adolescents [13]. This prevalence is high and may need further studies on existing suicidal behaviour.

In addition to the gap in the prevalence of suicidal behaviour in Saint Lucia, little is known regarding the context-specific predisposing and protective factors associated with suicidal behaviour in the country. Although Peltzer and Pengpid [13] noted that suicidal behaviour increased the odds of current alcohol use, having ever been drunk, and trouble from alcohol use among adolescents in this country, additional factors may be relevant in formulating policies and interventions to protect adolescents in Saint Lucia. In a recent study by Sarfo et al. [14] on the correlates of suicidal behaviour in Saint Vincent and the Grenadines, a closely related island, being male and having understanding parents or guardians were identified as protective factors against all suicidal behaviour (SI, SP, and SA). Additionally, Sarfo and co-workers identified risky adolescent behaviour such as truancy, violent behaviour, psychological problems, and substance use as risk factors for suicidal behaviour.

Similar to the findings of Sarfo et al. [14] and Peltzer and Pengpid [13], factors such as loneliness [4,15,16], alcohol use [16], and bullying [15,16,17] have been established as predictors of suicidal behaviour. As the world sets to meet the Sustainable Development Goals (SDG) by 2030, examining the prevalence and correlates of suicidal behaviour is crucial for the in-school adolescent population in Saint Lucia [18]. For example, SDG indicator 3.4.2 seeks to “reduce by one-third premature mortality from non-communicable diseases through prevention and treatment and promote mental health and well-being” by 2030, while SDG 4 aims to “ensure inclusive and equitable quality education and promote lifelong learning opportunities for all.” Thus, context-specific findings from the study would provide useful information for early risk detection and response to the existing picture of suicidal behaviour by adopting suicide prevention programmes for adolescents. 

Consequently, there is a need for each nation to enhance the thoroughness, accuracy, and timeliness of data on suicide, as suicide is becoming more common among adolescents globally [1]. Perhaps, adolescence may be a crucial “prevention window” for suicide [4,15]. Hence, the rationale of the present study is twofold: (1) to examine the prevalence of suicidal behaviour and (2) identify different correlates of SI, SP, and SA among nationally represented in-school adolescents in Saint Lucia. 

## 2. Materials and Methods

### 2.1. Study Design and Sample

The 2018 Global School Health Survey (GSHS) data on suicidal behaviour and correlates among school-aged adolescents (aged 13–17 years) from Saint Lucia were examined [19]. The GSHS is a school-based survey that employs a self-administered questionnaire to collect information on young people’s health behaviour and protective factors related to the leading causes of morbidity and mortality among adolescents and young adults of school age worldwide. The WHO carried out the GSHS in collaboration with the CDC of the United States (US) and the Saint Lucia Ministry of Health and Wellness. The study asked students in Saint Lucia to respond to close-ended structured questionnaires. The WHO website (https://extranet.who.int/ncdsmicrodata/index.php/catalog/877/related-materials, accessed on 10 March 2022) contains details of the systematic steps followed in collecting data from the respondents. 

### 2.2. Sampling Procedure

A two-stage cluster sample design was used to generate data representative of all Saint Lucian students in Forms 1–5, Lower 5, and Upper 6. All schools were chosen to participate in the first round. All classes, as were all students, were chosen to participate in the second stage. The response rate for the school was 100%, the response rate for students was 77%, and the overall response rate was 77%. The survey had 1864 students who responded to the study.

### 2.3. Study Measures

#### 2.3.1. Dependent Variables

Three primary outcome measures from the data on suicidal behaviour (SI, SP, and SA) were extracted. The study used a single self-report item or question to assess each of the suicidal behaviour (SI, SP, and SA). For example, SI was measured with the item, “During the past 12 months, did you ever seriously consider attempting suicide?” SP was measured with the question, “During the past 12 months, did you make a plan about how you would attempt suicide?”, and SA was measured by asking, “Did you actually attempt suicide in the last 12 months?” The responses to these questions were classified as “yes” or “no”. 

#### 2.3.2. Independent Variables

A set of independent variables, including participant demographics, psychological socio-environmental factors, and parental involvement, were analysed to explore how these variables predict the three outcome variables (SI, SP, and SA). Table 1 provides information about the questions, variable names, and coding used for the statistical analysis. 

#### 2.3.3. Ethical Statement

Before data collection began, the study received the necessary Institutional Review Board approval from the Saint Lucia Ministry of Health and Wellness and the Ministry of Education. Protocols for obtaining permission from the Ministry of Health and Wellness, the Ministry of Education, and the heads of the various schools involved in the study were followed. Furthermore, adolescents and parents of minors were asked to provide individual and parental informed consent (children below 18 years). 

### 2.4. Statistical Analysis

A sample weighting method was applied at the school, student, and sex levels within grades to make it representative of the adolescents in Saint Lucia to minimise bias on various trends of non-responses. Some variables were recorded on a binary scale in this study. We used the multiple imputations (MI) technique to address the issue of missing data. We applied the MI technique where the missing values exceeded 1%. The missing data ranged from 1% to 10% and were missing at random. 

First, all variables that have missing values were identified to evaluate the extent of missingness. Further, an imputation model was created for each variable with missing data prior to the MI. Five MIs with the automatic imputation method were conducted to maintain data quality concerning missing values. Imputed values were compared reasonably to observed values and results using the complete case analysis and additional sensitivity analysis. The results and the final model’s goodness of fit did not significantly differ from the original models following the MI procedure and data cleaning. The results revealed no evidence of a lack of fit with our model’s attempt to predict suicidal behaviour significantly. 

Additionally, a bivariate analysis using Pearson chi-square was performed to estimate the relationship between suicidal behaviour and the explanatory variables. We further entered the variables that showed significant association (*p* < 0.05) into a binomial logistic regression model after assessing the correlation between the independent variables to rule out the possibility of multicollinearity in the models. The results obtained from the analysis were presented with corresponding adjusted odds ratios (AOR) at a 95% confidence interval (CI) (*p* < 0.05). 

## 3. Results

### 3.1. Background Characteristics of Adolescents in Saint Lucia

**Figure 1:**
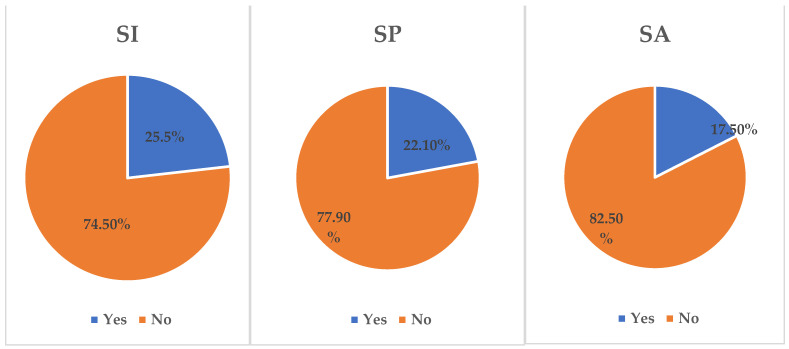
Prevalence of suicidal behaviour (SI, SP, and SA) among adolescents in St. Lucia.

From Figure 1, the prevalence rates of suicidal behaviour among the respondents were 25.5%, 22.1%, and 17.5% for SI, SP, and SA, respectively (see Figure 1). Significantly, more female adolescents had SI (18.4%), SP (36.7%), and SA (7.9%). Students who missed classes without permission significantly planned (5.4%) and attempted suicide (5.0%). Students who mostly or always felt hungry significantly had SI (3.1%) and SA (2.4%). Significantly, students who did not attend physical education (PE) classes experienced SI (11.7%). Moreover, adolescents who had used drugs such as amphetamine experienced SI (2.6%), had SP (2.6%), and attempted suicide (2.6%). More students who used marijuana significantly experienced SI (4.3%), made SP (4.3%), and attempted suicide (4.7%) than other participants who did not use it. 

Adolescents who drank alcohol and those who became drunk with alcohol significantly experienced SI (14.7%, 8.6%), made SP (13.2%, 8.2%), and attempted suicide (10.9%, 6.9%, respectively). More students who smoked cigarettes and those who used tobacco products other than cigarettes significantly experienced SI (4.9%, 4.0%), made SP (2.5%, 19.6%), and attempted suicide (3.6%, 3.3%, respectively). 

Likewise, students who were physically attacked experienced SI (9.7%), made SP (8.9%), and attempted suicide (7.6%). A significant number of students who engaged in physical fights had SI (9.7%), SP (8.7%), and attempted suicide (8.2%). Additionally, more students who were bullied and those who mostly or always felt lonely significantly experienced SI (10.1%, 10.1%), SP (9.1%, 9.0%), and attempted suicide (7.9%, 7.5%, respectively). More students who did not have close friends and those with multiple sexual partners significantly planned to commit suicide (3.6%, 5.6%) and attempted suicide (4.1%, 5.2%, respectively). Further, we found that students whose parents/guardians use tobacco and those who were mostly/always worried significantly experienced SI (5.6%, 8.2%), SP (4.5%, 6.8%), and attempted suicide (5.3%, 4.2%, respectively). Moreover, adolescents who attended PE classes on three or more days and those who attended PE classes on five or more days significantly experienced SI (5.4%, 3.4%, respectively) (see Table 2). 

### 3.2. Bivariate Analysis on the Association between Independent Variables and Suicidal Behaviour (SI, SP, and SA)

Bivariate analyses were used to determine the association between independent variables and suicidal behaviour (SI, SP, and SA). Sex of adolescents was significantly associated with SI (*p* < 0.001), SP (*p* < 0.001), and SA (*p* < 0.001). Significantly, adolescents who engaged in amphetamine use (*p* < 0.021, *p* < 0.001, *p* < 0.001), marijuana use (*p* < 0.032, *p* < 0.001, *p* < 0.001), alcohol abuse (*p* < 0.001, *p* < 0.001, *p* < 0.001), ever got drunk after consuming alcohol (*p* < 0.001, *p* < 0.001, *p* < 0.001), smoked cigarettes (*p* < 0.001, *p* < 0.001, *p* < 0.001), were physically attacked (*p* < 0.001, *p* < 0.001, *p* < 0.001), engaged in a physical fight (*p* < 0.0.024, *p* < 0.008, *p* < 0.001), were bullied (*p* < 0.001, *p* < 0.001, *p* < 0.001), felt lonely (*p* < 0.001, *p* < 0.001, *p* < 0.001), had parents who understood them (*p* < 0.001, *p* < 0.001, *p* < 0.001), had parents/guardians who used tobacco (*p* < 0.001, *p* < 0.001, *p* < 0.001), and were mostly/always worried (*p* < 0.001, *p* < 0.001, *p* < 0.001) were associated with the three suicidal behaviours (SI, SP, and SA, respectively). 

Adolescents who missed school without permission, those who did not have close friends, and those with multiple sexual partners were significantly associated with only SP (*p* < 0.002, *p* < 0.001, *p* < 0.003) and SA (*p* < 0.001, *p* < 0.001, *p* < 0.001, respectively). Adolescents who mostly/always experienced hunger were significantly associated with only SI (*p* < 0.030) and SA (*p* < 0.012), while attending PE classes for three or more days and five or more days was significantly associated with only SI (*p* < 0.015, *p* < 0.032, respectively) (see Table 2). 

### 3.3. Multivariate Regression Analysis on the Predictors of Suicidal Ideation, Planning, and Attempts

Table 3 shows the logistic regression for predictors of suicidal ideation, planning, and attempts. After adjusting for other factors associated with suicidal behaviour, males were less likely to have SI, SP, or SA in Saint Lucia. Moreover, adolescents who smoke cigarettes, currently use any tobacco products other than cigarettes, and do not have close friends added significance to only SA among respondents. 

Furthermore, being physically attacked significantly increased the odds of SI and SP only. Being bullied significantly increased the odds of SI, SP, and SA (AOR = 1.758, 95%CI = 1.308–2.363). Adolescents whose parents understood them were less likely to experience SI and SP only. Additionally, adolescents who mostly/always felt lonely and those who mostly worried about things they could not study were more likely to experience SI, SP, and SA (see Table 3).

## 4. Discussion 

This study, guided by the ITS, examined the prevalence and correlates of suicidal behaviour among school-going adolescents in Saint Lucia. The study found prevalence rates of 25.5%, 22.1%, and 17.5% for SI, SP, and SA, respectively. Comparatively, the prevalence of suicidal behaviour in Saint Lucia is relatively lower than the prevalence rates of 26%, 26%, and 19% for SI, SP, and SA in Saint Vincent and the Grenadines [14]. Additionally, the prevalence of SP among our study population is almost similar to that of a Ghanaian study which reported a 22.5% prevalence for SP [14]. Still, the prevalence of SI and SA was higher in the present study than in the Ghanaian study. For all three suicidal behaviours among the study sample, the prevalence rates are relatively higher than those reported among adolescents in the USA [20,21] and Vietnam [22]. Additionally, the prevalence rates of SI and SP are higher than the findings of a study in Mozambique [23], except for a reported 18.5% prevalence of SA, a figure slightly higher than the present observation. Differences in the sample sizes, cultures, and study periods may account for the variations in the reported prevalence rates. Nevertheless, the current prevalence rates show how problematic suicide is among adolescents in Saint Lucia. This evidence raises concern for school-based interventions to help address triggers of suicidal behaviour among adolescents in Saint Lucia to attain the global SDG indicators of suicide prevention [18]. It is evident that the factors associated with the suicidal rate may need a multidisciplinary approach and policy to deal with.

Contrary to previous studies [4,17,18], sex was identified as a major predictor of all three indices of suicidal behaviour. Being a male was associated with lower odds of SI, SP, and SA among adolescents in Saint Lucia. This outcome is consistent with previous studies, which upheld that suicidal behaviour is less pronounced among males than females, implying that females are more predisposed to suicidal behaviour [5,16,24]. The “gender paradox” posits different perspectives that explain gender differences and the risk of suicidal behaviour [25]. Previous studies have reported that female adolescents are more likely to engage in SI and SA than their male counterparts, partially because of higher depression levels [26,27]. Hence, internalising disorders could mediate the high risk of suicidal behaviour among female adolescents (e.g., depression, anxiety), whose effects are much more telling in adolescent girls [28,29]. Other scholarly information suggests that females are more likely to be suicidal because of relationship problems compared to men in response to socioeconomic crises [30]. Therefore, context-specific norms, beliefs, and patterns may determine sex and suicidal behaviour, which may vary across cultures due to diverse femininity and masculinity cultural scripts [25,31]. This finding iterates the need for female-specific strategies to be incorporated into school-based programs to help prevent suicidal behaviour among female adolescents in Saint Lucia. 

Adolescents in Saint Lucia who smoked cigarettes or used tobacco products other than cigarettes were twice as likely to attempt suicide compared to peers who did not use any of these products. There is sufficient empirical evidence on the association between cigarette smoking, tobacco use, and the risk of suicidal behaviour [16,24,32,33,34], yet the mechanisms underlying the use of these substances and suicide remain vague. Nonetheless, adolescents are more prone to nicotine-induced injuries due to the developing nature of their brains. Nicotine, as a potent activator of the hypothalamic–pituitary–adrenal (HPA) axis [35], triggers hyperactivity of the HPA, especially among adolescents, which predisposes them to suicidal behaviour. The HPA axis is known to play a significant role in suicide risk [36]. Second, cigarette smoking, tobacco use, and other covariates such as risky and violent behaviour (e.g., sexual abuse, physical abuse, and alcohol use) are already established risk factors for suicidal behaviour and may serve as pathways to adolescents’ suicidal behaviour. A key lesson from this finding is that schools in Saint Lucia are encouraged consider substance use (especially tobacco and cigarettes) when assessing school children for suicide risk in order to develop appropriate interventions (e.g., smoking cessation therapy). 

In Saint Lucia, psychosocial factors such as physical attacks and bullying were significantly associated with suicidal behaviour. Adolescents who fell victim to physical attacks were at higher risk of ideating and planning suicide than their peers who were not physically attacked. Moreover, victims of bullying had increased odds of SI, SP, and SA. The association between physical attacks, bullying, and suicidal behaviour is globally evident [4,5,16,23,37]. Using the IPTS as a guide, most physically attacked and bullied adolescents may be unable to stand these treatments’ humiliation [38,39]. This feeling of being humiliated breeds arrays of psychologically distressing symptoms, especially internalising disorders (e.g., anxiety and depression), which have already been established as major predictors of suicidal behaviour [40,41,42]. A supportive approach through integrating rehabilitative interventions for school children who fall victim to physical attacks and bullying could be pivotal in preventing suicidal behaviour among adolescents in Saint Lucia. 

After adjusting for other factors, a significant association was found between parent/guidance tobacco use and self-reported SI, but not other suicidal behaviour such as SA. Contrary to this finding, a study in the United States found that teens whose parents do not smoke have increased odds of SI [43]. The same study reported that there is no significant risk for SI among teens with parents who smoke [43]. In previous studies, however, parental use of tobacco has been found as a predictor of SA [44,45]. Considering the contrasting evidence on parental tobacco use and SI, further studies are required to investigate this link. Nonetheless, we speculate that since parents who use tobacco and other substance may perpetrate domestic violence against children [46], it is possible that adolescents who fall victim to domestic violence may end up ideating suicide due to the severity of damage they may have experienced from such parents. 

Supporting the finding of Oppong Asante et al. [5], adolescents who had close friends were more likely to plan suicide and had three times higher odds of SA compared to peers without close friends. However, previous studies contradict the present study finding, which revealed that having no close friends rather increased the odds of suicidal behaviour among adolescents [4,45]. Despite the discrepancies, it is assumed that the nature of friendships and their impact on an adolescent might be a factor to consider in explaining this link. Possibly, delinquent and aggressive friends may serve as perpetrators of SP and SA. Friends may also play a role in reviewing failed SP and SA by suggesting alternative approaches. Nevertheless, close friends of adolescents who plan and attempt suicide may lack the support to dissuade them from such suicidal behaviour. 

For SI, SP, and SA, loneliness was found as a key psychosocial predictor that increased the odds by more than two among the study population. Thus, adolescents who felt lonely had higher odds of ideating, planning, and attempting suicide in Saint Lucia. Clearly, loneliness as a major predictor of suicidal behaviour has been unequivocally reported by previous studies [16,47,48]. Sometimes, adolescents’ feelings of loneliness may not directly imply that they do not have people around them. This situation is because loneliness is characterised by a subjective perception of not being in touch with people [49]. The thwarted belongingness perspective suggests that although adolescents may have peers, family, or community members around them, the feeling of loneliness may be triggered by the extent of interactions and bonds between them and others around them. With a weak bond, adolescents may feel rejected or disliked by others, hence the ideation, planning, and attempt of suicide [7,8]. 

Adolescents who were worried had higher odds of ideating, planning, and attempting suicide than peers who did not get worried. Similarly, Law and Tucker [50] found that worrying increases the odds of suicide among people who are earlier predisposed to SI. Although not many studies have clearly stated the role of worry in suicidal behaviour, we have a number of plausible explanations to support this study’s finding. Worry is a state of anxiety. This anxiety dimension has already been shown to be a predictor of suicidal behaviour [51,52]. Among school-going adolescents, academic-related anxiety is most prevalent due to their desire to meet academic demands [53]. This finding suggests the need for Saint Lucia schools to prioritise providing children with mental health services. 

In previous studies, adolescents with understanding and supportive parents were protected from suicidal behaviour [5,16]. Among the studied sample, SI, SP, and SA were less likely among adolescents with understanding parents. Parental influence on the well-being of adolescents cannot be understated. Due to their development, adolescents require maximum support from parents, which comes through the showing of love, compassion, and care. In moments of distress, adolescents may be able to confide in their parents on the basis that these parents understand enough. This may bolster confidence and resound hope in adolescents, decreasing their likelihood to ideate, plan, or attempt suicide. Taken from the thwarted belongingness dimension, maladaptive intra (e.g., loneliness, worry) and interpersonal reactions (e.g., low-income family climate and communication, parental neglect and insecure parent–child attachment, increased aggression, reduced helpfulness) may cause a decline in the quality of interpersonal interactions and trigger suicidal behaviour [54,55].

Aside from the demographic and drug and substance use correlates, our study acknowledges the role of several psychosocial factors as predictors of suicidal behaviours based on the ITS [7]. Emerging evidence suggests that many of these variables can be incorporated into the construct of demoralisation. Drawing from the pioneering work by Clarke and Kissane [56], the concept of demoralisation, which manifests as existential despair, hopelessness, helplessness, and a loss of meaning and purpose in life, has frequently been seen in patients who are medically and psychiatrically unwell. In support of this, a systematic review of 18 studies showed that demoralisation could be linked to suicidal behaviour in various populations, including patients with somatic or mental problems and community residents, and can considerably increase the risk of suicide [57]. Though this concept is yet to gain widespread attention within the study of suicidality and based on Costanza et al.’s [57] findings, determining demoralisation may aid in a more thorough assessment of suicide risk among school-going adolescents in Saint Lucia. 

### 4.1. Strengths and Limitations

The study used a national dataset to investigate suicidal behaviour among adolescents in Saint Lucia. The sample’s representativeness allows us to learn more about the factors that increase the likelihood that these adolescents in Saint Lucia would experience suicidal behaviour. On the other hand, since the GSHS does not have data on adolescents not in education, the study focused primarily on school-going adolescents in Saint Lucia, without considering those out of school who may have had higher probabilities of engaging in suicidal and risky behaviours. Given that the GSHS is cross-sectional, a causal link between the several factors and suicidal behaviour cannot be established. Additionally, a single item was used to measure several mental health dimensions, such as bullying, worrying, and suicidal behaviour (SI, SP, and SA). The item construction may not fully capture all clinical symptoms for diagnostic purposes. Again, suicidal behaviour was assessed based on a 12-month period prior to the survey, thus undermining the prevalence of lifetime suicidal behaviour. Moreover, a previous SA and depression, which are important risk factors for suicide, could not be examined as predictor variables in this study since there were no data on them in the GSHS. Despite these limitations, the study findings serve as a foundation for subsequent research and interventions involving in-school adolescents in Saint Lucia. 

### 4.2. Practical Implications 

The findings consider the psychosocial, personal, and demographic characteristics connected to suicidal behaviour among in-school students. Based on the assumptions of the ITS [7] and findings, school authorities in Saint Lucia ought to offer mental health services and support mechanisms for students through behaviour monitoring, direction, and counselling on stress management and how to react to physical behaviours and bullying [58]. It would be useful to designate a few members of the school staff as mental health focal points or call points and teach them the fundamental skills for spotting pupils who are most vulnerable to suicidal behaviour [5]. Additionally, through schools, Saint Lucia’s educational system is encouraged to develop suicidal behavioural risk assessment tools available through digital platforms, where children can answer standardised questionnaires about suicidal behaviour during predetermined times. Drawing from the ITS and study recommendations of Costanza et al. [59], digital platforms that inspire psychological models and clinical prevention approaches can be modified to prevent suicidal behaviour within the context of school-going adolescents. Finding each student’s risk factors for suicidal behaviour and advising proper management and referrals in light of those risks would help manage this problem in schools [60]. 

Adolescents’ use of drugs and other substances could be a key area of attention for suicidal behaviour prevention initiatives. Particularly, amphetamine, marijuana, cigarettes, tobacco, and alcohol usage have been linked to many types of suicidal behaviour. Preventing student substance usage through cognitive-behavioural interventions (e.g., alcohol and smoking cessation therapy training) designed and implemented in the schools would have a positive effect on both academic performance and aggression levels. Creating a supportive school climate or environment that offers several opportunities for skill development and training through creative arts, athletics, and other lifelong pursuits that pique adolescents’ interests could be very useful and serve as a substitute for social vices. These goals can be achieved through establishing cooperative efforts between policymakers, the school, and other pertinent stakeholders to address social and behavioural issues affecting in-school adolescents in the country. These attempts will significantly help reduce suicidal behaviour, improve mental health, and improve academic outcomes. Parental connectedness through communication, monitoring, and family support systems may significantly manage suicidal behaviour among in-school adolescents in Saint Lucia.

## 5. Conclusions

The study discovered a relatively high prevalence of suicidal behaviour, influenced by multiple intra-and interpersonal factors among in-school adolescents in Saint Lucia, using nationally representative data from the 2018 GSHS. Identifying proactive steps to lower the prevalence of suicidal behaviour will assist Saint Lucia in achieving some of the SDG targets, particularly SDG indicators 3.5 and 4.1 (strengthen the prevention and treatment of substance abuse, including narcotic drug abuse and harmful alcohol use; ensure inclusive and equitable quality education, and promote opportunities for lifelong learning for all). Thus, collaborative efforts from the government, schools, parents, and other stakeholders could help strengthen policies and programs to influence adolescent behaviour in schools across Saint Lucia.

## Figures and Tables

**Table 1:** Definition of explanatory variables.

Explanatory Variables	Questions
Sex	What is your sex?
Age	How old are you?
Grade	What grade are you in?
School truancy	In the past 30 days, did you miss classes or school without permission?
Hunger	Were you hungry most of the time, or did you always go hungry?
Missed PE classes	Did you miss the recent physical education classes?
Physical attack	Have you been attacked physically before?
Suicidal ideation	In the past 12 months, did you ever seriously consider attempting suicide?
Suicide attempt	In the past 12 months, did you attempt suicide?
Suicide plan	In the past 12 months, did you make a plan about how you would attempt suicide?
School truancy	In the past 30 days, did you miss classes or school without permission?
Amphetamine use	In your life, did you use amphetamine or methamphetamine (also called ice or yellow)?
Current use of alcohol	In the past 30 days, did you have at least one drink containing alcohol?
Ever got drunk after consuming alcohol	Have you ever drunk so much alcohol that you were really drunk?
Marijuana smoking	In the past 30 days, did you use marijuana?
Cigarette smoking	Do you currently smoke cigarettes?
Use of tobacco products other than cigarette	Do you use any other tobacco product apart from cigarettes?
Parental use of tobacco	Do you have parents or guardians who use any form of tobacco?
Physical fight	Have you engaged in a physical fight before?
Bullied	Have you been bullied at school in the past 12 months?
Attended physical education classes on ≥3 days	Did you attend physical education classes on three or more days?
Attended physical education classes on ≥5 days	Did you attend physical education classes on five or more days?
Close friends	Do you have close friends?
Loneliness	Do you feel lonely most of the time or always?
Worry	Do you most of the time or always worry about something that you could not study?
Sex with multiple sexual partners	Have you slept with two or more partners before?

**Table 2:** Bivariate analysis between independent variables and suicidal behaviour (SI, SP, and SA).

*Variables*		Suicidal Ideation (N = 1864)			Suicide Plan (N = 1864)			Suicide Attempt (N = 1864)		
		Yes	No	Chi-Square (χ^2^)	Yes	No	Chi-Square (χ^2^)	Yes	No	Chi-Square (χ^2^)
***Demographic***										
Age (years)	12–14	247 (13.3%)	740 (39.7%)	0.29	219 (11.7%)	768 (41.2%)	0.01	180 (9.7%)	807 (43.3%)	0.70
	15–17	229 (12.3%)	648 (34.8%)		684 (36.7%)	193 (10.4%)		147 (7.9%)	730 (39.2%)	
Sex	Male	133 (7.1%)	732 (39.3%)	87.63 ***	129 (6.9%)	736 (39.5%)	48.46 ***	121 (6.5%)	744 (39.9%)	14.08 ***
	Female	343 (18.4%)	656 (35.2%)		283 (15.2%)	716 (38.4%)		206 (11.1%)	793 (42.5%)	
Grade	1–3	237 (12.7%)	728 (39.1%)	1.00	210 (11.3%)	755 (40.5%)	0.135	183 (9.8%)	782 (42.0%)	2.79
	4–6	239 (12.8%)	660 (35.4%)		202 (10.8%)	697 (37.4%)		144 (7.7%)	755 (40.5%)	
***Personal***										
Truancy	Yes	104 (5.6%)	254 (13.6%)	2.88	101 (5.4%)	257 (13.8%)	9.61 **	94 (5.0%)	264 (14.2%	23.26 ***
	No	372 (20.0%)	1134 (60.8%)		311 (16.7%)	1195 (64.1%)		233 (12.5%)	1273 (68.3%)	
Hunger	Yes	58 (3.1%)	120 (6.4%)	5.14 *	46 (2.5%)	132 (7.1%)	1.60	44 (2.4%)	134 (7.2%)	7.01 *
	No	418 (22.4%)	1268 (68.0%)		366 (19.6%)	1320 (70.8%)		283 (15.2%)	1403 (75.3%)	
Missed PE classes	Yes	218 (11.7%)	563 (30.2%)	3.99 *	180 (9.7%)	601 (32.2%)	0.70	130 (7.0%)	651 (34.9%)	0.75
	No	258 (13.8%)	825 (44.3%)		232 (12.4%)	851 (45.7%)		197 (10.6%)	886 (47.5%)	
***Drug and substance use***										
Amphetamine or methamphetamines use	Yes	48 (2.6%)	93 (5.0%)	5.80 *	49 (2.6%)	92 (4.9%)	14.17 ***	55 (3.0%)	86 (4.6%)	48.58 ***
	No	428 (23.0%)	1295 (69.5%)		363 (19.5%)	1360 (73.0%)		272 (14.6%)	1451 (77.8%)	
Marijuana use	Yes	81 (4.3%)	179 (9.6%)	5.01 *	80 (4.3%)	180 (9.7%)	13.18 **	87 (4.7%)	173 (9.3%)	52.93 ***
	No	395 (21.2%)	1209 (64.9%)		332 (17.8%)	1272 (68.2%)		240 (12.9%)	1364 (73.2%)	
Drunk alcohol	Yes	274 (14.7%)	598 (32.1%)	29.85 ***	246 (13.2%)	626 (33.6%)	35.50 ***	203 (10.9%)	669 (35.9%)	37.28 ***
	No	202 (10.8%)	790 (42.4%)		166 (8.9%)	826 (44.3%)		124 (6.7%)	868 (46.6%)	
Ever got drunk after consuming alcohol	Yes	160 (8.6%)	324 (17.4%)	19.45 ***	152 (8.2%)	332 (17.8%)	32.85 ***	128 (6.9%)	356 (19.1%)	35.83 ***
	No	316 (17.0%)	316 (17.0%)		260 (13.9%)	1120 (60.1%)		199 (10.7%)	1181 (63.4%)	
Smoke cigarettes	Yes	57 (3.1%)	92 (4.9%)	13.78 ***	59 (3.2%)	90 (4.8%)	28.79 ***	68 (3.6%)	81 (4.3%)	88.37 ***
	No	419 (22.5%)	1296 (69.5%)		353 (18.9%)	1362 (73.1%)		259 (13.9%)	1456 (78.1%)	
Use of tobacco products other than cigarette	Yes	46 (2.5%)	75 (4.0%)	10.60 **	47 (2.5%)	74 (4.0%)	21.06 ***	61 (3.3%)	60 (3.2%)	96.65 ***
	No	430 (23.1%)	430 (23.1%)		365 (19.6%)	1378 (73.9%)		266 (14.3%)	1477 (79.2%)	
***Psychosocial***										
Physically attacked	Yes	181 (9.7%)	449 (24.1%)	22.87 ***	166 (8.9%)	393 (21.1%)	26.74 ***	142 (7.6%)	417 (22.4%)	34.10 ***
	No	295 (15.8%)	939 (50.4%)		246 (13.2%)	1059 (56.8%)		185 (9.9%)	1120 (60.1%)	
Physical fight	Yes	181 (9.7%)	449 (24.1%)	5.10 *	162 (8.7%)	468 (25.1%)	7.21 **	152 (8.2%)	478 (25.6%)	28.52 ***
	No	295 (15.8%)	939 (50.4%)		250 (13.4%)	984 (52.8%)		175 (9.4%)	1059 (56.8%)	
Bullied	Yes	188 (10.1%)	283 (15.2%)	68.52 ***	170 (9.1%)	301 (16.1%)	71.65 ***	147 (7.9%)	324 (17.4%)	81.39 ***
	No	288 (15.5%)	1105 (59.3%)		242 (13.0%)	1151 (61.7%)		180 (9.7%)	1213 (65.1%)	
Loneliness	Yes	189 (10.1%)	177 (9.5%)	163.18 ***	167 (9.0%)	199 (10.7%)	146.39 ***	140 (7.5%)	226 (12.1%)	135.02 ***
	No	287 (15.4%)	177 (9.5%)		245 (13.1%)	1253 (67.2%)		187 (10.0%)	1311 (70.3%)	
Close friends	Yes	64 (3.4%)	150 (8.0%)	2.43	68 (3.6%)	146 (7.8%)	13.14 ***	76 (4.1%)	138 (7.4%)	53.98 ***
	No	412 (22.1%)	1238 (66.4%)		344 (18.5%)	1306 (70.1%)		251 (13.5%)	1399 (75.1%)	
Parents/guardians’ use of tobacco	Yes	104 (5.6%)	158 (8.5%)	32.14 ****	83 (4.5%)	179 (9.6%)	16.24 ***	79 (4.2%)	183 (9.8%)	33.51 ***
	No	372 (20.0%)	1230 (66.0%)		329 (17.7%)	1273 (68.3%)		248 (13.3%)	1354 (72.6%)	
Understanding parents	Yes	113 (6.1%)	554 (29.7%)	40.35 ***	98 (5.3%)	569 (30.5%)	33.13 ***	81 (4.3%)	586 (31.4%)	20.93 ***
	No	363 (19.5%)	834 (44.7%)		314 (16.8%)	883 (47.4%)		246 (13.2%)	951 (51.0%)	
Multiple sexual partners	Yes	107 (5.7%)	266 (14.3%)	2.43	104 (5.6%)	269 (14.4%)	9.05 **	96 (5.2%)	277 (14.9%)	21.65 ***
	No	369 (19.8%)	1122 (60.2%)		308 (16.5%)	1183 (63.5%)		231 (12.4%)	1260 (67.6%)	
Attended PE classes on ≥3 days	Yes	101 (5.4%)	373 (20.0%)	5.98 *	95 (5.1%)	379 (20.3%)	1.57	82 (4.4%)	392 (21.0%)	0.03
	No	375 (20.1%)	1015 (54.5%)		317 (17.0%)	1073 (57.6%)		245 (13.1%)	1145 (61.4%)	
Attended PE classes on ≥5 days	Yes	64 (3.4%)	246 (13.2%)	4.68 *	56 (3.0%)	254 (13.6%)	3.52	44 2.4%)	266 (14.3%)	2.88
	No	412 (22.1%)	1142 (61.3%)		356 (19.1%)	1198 (64.3%)		283 (15.2%)	1271 (68.2%)	
Worried	Yes	152 (8.2%)	138 (7.4%)	130.47 ***	126 (6.8%)	164 (8.8%)	90.88 ***	118 (6.3%)	172 (9.2%)	127.20 ***
	No	324 (17.4%)	1250 (67.1%)		286 (15.3%)	1288 (69.1%)		209 (11.2%)	1365 (73.2%)	

Note: * *p* < 0.05, ** *p* < 0.01, *** *p* < 0.001.

**Table 3:** Logistic regression for predictors of SI, SP, and SA.

Variable	Suicidal Ideation	Suicide Plan	Suicide Attempt
	AOR (95%CI)	AOR (95%CI)	AOR (95%CI)
***Demographic***			
Sex	0.324(0.248–0.422) ***	0.365(0.276–0.483) ***	0.453(0.330–0.621) ***
***Personal***			
Truancy	–	1.181(0.866–1.611)	1.249(0.891–1.751)
Hunger	0.879(0.593–1.303)	–	0.713(0.454–1.119)
Missed PE classes	0.995(0.763–1.296)	–	–
***Drug and substance use***			
Amphetamine or methamphetamines use	1.151(0.698–1.899)	1.247(0.767–2.028)	1.380(0.830–2.295)
Marijuana use	1.063(0.727–1.554)	1.030(0.699–1.516)	1.325(0.881–1.992)
Alcohol	1.358(1.057–1.744)	1.371(1.056–1.781)	1.339(0.997–1.799)
Ever got drunk after consuming alcohol	1.146(0.855–1.534)	1.266(0.940–1.705)	0.953(0.683–1.330)
Smoke cigarettes	1.226(0.764–1.968)	1.504(0.948–2.386)	2.092(1.297–3.374) ** (0.002)
Currently used any tobacco products other than cigarettes	1.002(0.593–1.695)	1.052(0.630–1.755)	2.055(1.226–3.443) ** (0.006)
***Psychosocial***			
Physically attacked	1.425(1.087–1.868) *	1.367(1.039–1.800) *	1.245(0.919–1.689)
Physical fight	1.080(0.823–1.416)	1.043(0.790–1.378)	1.261(0.929–1.711)
Bullied	1.671(1.287–2.169) ***	1.716(1.315–2.240) ***	1.758(1.308–2.363) ***
Parents/guardians’ use of tobacco	1.637(1.195–2.244) **	0.308(1.188–0.853)	1.412(0.987–2.019)
Understanding parents	0.627(0.482–0.816) ***	0.662(0.504–0.869) **	0.800(0.588–1.089)
Multiple sexual partners	–	1.336(0.959–1.860)	1.402(0.978–2.009)
Close friends	–	1.587(1.118–2.253) *	3.021(2.102–4.341) ***
Loneliness	2.634(1.999–3.471) ***	2.649(2.006–3.498) ***	2.669(1.961–3.632) ***
Attended PE classes on ≥3 days	0.764(0.503–1.159)	–	–
Attended PE classes on ≥5 days	1.016(0.637–1.621)	–	–
Worry	2.375(1.757–3.210) ***	1.703(1.251–2.319) **	2.347(1.687–3.265) ***
Constant	0.056 **		

Note: * *p* < 0.05, ** *p* < 0.01, *** *p* < 0.001. Hosmer and Lemeshow test (goodness of fit), SI [χ^2^ (8) = 10.430, *p* = 0.235]; SP [χ^2^ (8) = 5.184, *p* = 0.738], SA [χ^2^ (8) = 2.261, *p* = 0.972].

## Data Availability

This paper uses data from the GSHS database. The WHO and the CDC supported the GSHS data, freely available at: https://extranet.who.int/ncdsmicrodata/index.php/catalog/878, accessed on 10 March 2022.
